# Visualization of *C. elegans *transgenic arrays by GFP

**DOI:** 10.1186/1471-2156-7-36

**Published:** 2006-06-07

**Authors:** Aidyl S Gonzalez-Serricchio, Paul W Sternberg

**Affiliations:** 1 Department of Biological Sciences, California State Polytechnic University, 3801 W Temple Avenue, Pomona, CA 91768, USA; 2 Division of Biology and Howard Hughes Medical Institute, mail code 156-29, Caltech, Pasadena, CA 91125, USA

## Abstract

**Background:**

Targeting the green fluorescent protein (GFP) via the *E. coli lac *repressor (LacI) to a specific DNA sequence, the *lac *operator (*lacO*), allows visualization of chromosomes in yeast and mammalian cells. In principle this method of visualization could be used for genetic mosaic analysis, which requires cell-autonomous markers that can be scored easily and at single cell resolution. The *C. elegans lin-3 *gene encodes an epidermal growth factor family (EGF) growth factor. *lin-3 *is expressed in the gonadal anchor cell and acts through LET-23 (transmembrane protein tyrosine kinase and ortholog of EGF receptor) to signal the vulval precursor cells to generate vulval tissue. *lin-3 *is expressed in the vulval cells later, and recent evidence raises the possibility that *lin-3 *acts in the vulval cells as a relay signal during vulval induction. It is thus of interest to test the site of action of *lin-3 *by mosaic analysis.

**Results:**

We visualized transgenes in living *C. elegans *by targeting the green fluorescent protein (GFP) via the *E. coli lac *repressor (LacI) to a specific 256 sequence repeat of the *lac *operator (*lacO*) incorporated into transgenes. We engineered animals to express a nuclear-localized GFP-LacI fusion protein. *C. elegans *cells having a *lacO *transgene result in nuclear-localized bright spots (i.e., GFP-LacI bound to *lacO*). Cells with diffuse nuclear fluorescence correspond to unbound nuclear localized GFP-LacI. We detected chromosomes in living animals by chromosomally integrating the array of the *lacO *repeat sequence and visualizing the integrated transgene with GFP-LacI.

This detection system can be applied to determine polyploidy as well as investigating chromosome segregation. To assess the GFP-LacI•*lacO *system as a marker for mosaic analysis, we conducted genetic mosaic analysis of the epidermal growth factor *lin-3*, expressed in the anchor cell. We establish that *lin-3 *acts in the anchor cell to induce vulva development, demonstrating this method's utility in detecting the presence of a transgene.

**Conclusion:**

The GFP-LacI•*lacO *transgene detection system works in *C. elegans *for visualization of chromosomes and extrachromosomal transgenes. It can be used as a marker for genetic mosaic analysis. The *lacO *repeat sequence as an extrachromosomal array becomes a valuable technique allowing rapid, accurate determination of spontaneous loss of the array, thereby allowing high-resolution mosaic analysis. The *lin-3 *gene is required in the anchor cell to induce the epidermal vulval precursors cells to undergo vulval development.

## Background

The green fluorescent protein (GFP) of the jellyfish *Aequorea victoria *has been used extensively for observation *in vivo *of gene expression and cell morphology in *C. elegans *[1-4]. GFP has also been targeted to specific subcellular structures by fusing GFP to various proteins. A technique utilizing a chimeric protein of GFP (S65T) and the *E. coli *lac repressor (LacI) along with lac operator (*lacO*) makes the visualization of chromosomes possible [5-8]. This fusion protein has the DNA-binding capability of LacI and the fluorescent properties of GFP. The fusion protein is capable of binding to the *lacO*, thus localizing GFP expression at the DNA repeat. Such localization allows direct visualization of segregating chromosomes during mitosis.

We have applied the GFP-LacI technique to *C. elegans*. We show that the GFP-LacI•*lacO *repeat technique allows visualization of transgenes present as either extrachromosomal arrays or integrated into a chromosome. The integrated version allows visualization of chromosomal segregation and determination of polyploidy.

Visualization of extrachromosomal arrays provides a method to detect transgenes used for mosaic analysis. Genetic mosaics in *C. elegans *are typically generated by the spontaneous somatic loss of an extrachromosomal transgenic array or a free duplication [9-13]. When the free duplication or extrachromosomal array containing a wild-type cell-autonomous marker gene (often *ncl-1; *enlarged nucleoli) and a gene of interest is lost from one of the daughter cells during mitosis, it gives rise to a lineage of cells lacking wild-type activity of the marker gene and of the gene of interest. Extra-chromosomal arrays are mitotically unstable, resulting in a complex mosaic pattern, establishing a method of scoring individual cells under Nomarski differential interference contrast microscopy.

The inductive signal for hermaphrodite vulval differentiation is the epidermal growth factor (EGF) like protein LIN-3 [14,15]. *lin-3 *encodes proteins that have an extracellular domain with one EGF motif, a transmembrane domain and a cytoplasmic domain. In the presence of the gonadal anchor cell (AC), three of the six vulva precursor cells (VPCs) undergo three rounds of mitosis and generate the cells that form the vulva. The VPCs are the posterior daughters (P3.p-P8.p) of six of the twelve Pn cells present at hatching [16,17]. The VPC (P6.p) nearest to the AC will adopt the 1° fate since it receives more signal than its neighbors. P5.p and P7.p cells are induced to adopt the 2° fate, either directly by LIN-3 [18] or indirectly via the 1° VPC [19]. The VPCs (P3.p, P4.p. and P8.p) further from the AC adopt the 3° fate, which is to generate two non-vulval descendants that fuse with the hyp7 epidermal syncytium. The fates adopted by the VPCs are distinguished in part by the number of progeny they generate. The 1° and 2° cell fates generate eight and seven descendants, respectively, which form the mature vulva [15]. Decreased *lin-3 *activity results in decreased vulval development while overexpression of *lin-3 *results in increased vulval development. Genetic epistasis tests indicated that *lin-3 *acts upstream of *let-23*, *sem-5*, *let-341*, *let-60, lin-45*, *mek-2 *and *mpk-1 *during vulval induction [reviewed by [20]]. Based upon *lacZ *and GFP reporter gene constructs, *lin-3 *is expressed in the anchor cell at the time of vulval induction [14,21-23], and in the 1° vulval lineage after vulval induction [24]. Recently, Dutt et al. [25] argue based on molecular genetic experiments that *lin-3 *can act in the VPCs to extend the range of induction. Based upon its structure, expression and genetic properties, the AC has been proposed to secrete LIN-3 protein [14,18]. We have tested by mosaic analysis, using GFP-LacI•*lacO*, whether *lin-3 *signal is required solely in the anchor cell for vulval induction.

## Results and discussion

### Visualization of chromosomes

To test whether the GFP-LacI•*lacO *system could be used to visualize the DNA of extrachromosomal arrays in *C. elegans*, we engineered sequences that encode a GFP-LacI fusion protein under the control of the heat-shock enhancer/promoter in vector pPD49-78 (hsGFP-LacI). We then microinjected a DNA mixture containing hsGFP-LacI, the *lacO *repeat and *dpy-20 *rescuing DNA (pMH86) into the gonad of an adult *dpy-20(e1282) *hermaphrodite. After a 30-minute heat-shock at 33°C, transformants were found to express nuclear GFP and have intense foci of subnuclear fluorescence, presumably corresponding to the DNA of the extrachromosomal arrays. DAPI co-staining confirmed that the GFP-LacI•*lacO *system has nuclear expression and association with DNA. We first detected expression in embryos at early gastrulation (~24 cell stage). Larvae and adults express GFP broadly.

**Figure 1:**
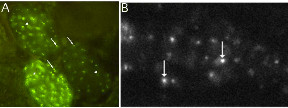
**Visualization of *C. elegans *extrachromosomal arrays by GFP**. One to two bright spots are seen per nucleus when the fusion proteins are bound to the *lacO *array. If the fusion proteins remain unbound, then the nucleus has diffuse fluorescence (arrowheads). If fusion proteins bind to the *lacO *array then the nucleus have localized bright spots (arrows). (A) Embryos: *dpy-20; syEx *[pMH86, pPD49-78::GFP-LacI, *lacO*] embryos were mounted on 5% Noble agar and examined under Nomarski optics and epifluorescence at 100 X. (B) A confocal photomicrograph of a *syIs44 *L4 hermaphrodite pharynx.

DNA molecules injected into the *C. elegans *gonad syncytium assemble into arrays; extrachromosomal arrays consist of many rearrangements of the DNA injected [26]. The fusion protein bound to the *lacO *repeat resulting in one to two bright spots per nucleus as well as the unbound fusion protein resulting in nuclear diffuse fluorescence ("haze") (Figure 1). Mitotic loss of these mixed extrachromosomal arrays in a single founder cell results in a clone of cells lacking the activities of all genes in the array [11,27].

To test the reliability and consistency of the GFP-LacI•*lacO *as a detection method for transgenes, we examined animals in which every cell has the *lacO *array and in which most cells express GFP-LacI. Specifically, non-Dpy transformants of *dpy-20(e1282); syEx [pMH86 + pPD49-78GFP-LacI + lacO] *were X-irradiated to integrate the transgenic array into the genome, yielding integrated transgene *syIs44 *in the strain PS2442 (Table 1). In this strain, we observed that most nuclei had one or more spots of fluorescence (see Figure 1B). Detection of chromosomes was efficient. For example, we observed two pairs of sister cells in each of the 20 animals, the embryonic sisters F and U and the postembryonic sisters P8.pa and P8.pp. We found that all 80 cells had one or two fluorescent spots. Therefore, we are able to detect a transgene in every cell.

**Table 1:** Transgenes and strains

PS2442: *dpy-20(e1282;) syIs44 *[pMH86 (*dpy-20(+)*), pPD49-78GFP-lacI, *lacO*] [strain available from the CGC]
PS2381: *ncl-1(e1865) II; syEx154 *[pRF4 *(rol-6(su1006)*), cosmid C33C3, pPD49-78GFP-lacI, *lacO*]
PS2629: *lin-5(e1348), dpy-10(e128) II*; *syEx207 *[pRF4 *(rol-6(su1006)*), pPD49-78GFP-LacI, *lacO*_*256*_]
PS2958: *syIs46 *[pMH86 (*dpy-20(+)*), dpy-30S65T*LacI, pPD49-78GFP-LacI] *II; ncl-1(e1865) III; dpy-20(e1282) IV; him-5(e1490) V *[strain available from the CGC]
PS3047: *syIs46; ncl-1(e1865); dpy-20(e1282); lin-15(e1763); syEx272 *[*lin-15B*(+), *lacO*, cosmid C33C3]
PS3427: *syIs46; lin-3(n378) let-59(s49) unc-22(s7)/lin-3(n1059) unc-24(e138); syEx345 *[*lin-3*(+), *lacO*, pPD118-33]

We then examined the behavior of an extrachromosomal transgene carrying *lacO *in a strain (PS3427) that expressed GFP-LacI from an integrated trasngene (*syIs46*). To ascertain whether the loss of the array coincides with the progenitor cell and its two daughters, we looked at the nuclei of two sister cells, namely, F and U (Table 2). Of the 67 animals viewed with the integrated GFP-LacI and the extrachromosomal *lacO*, 65.6% of the progenitor cells had a localized bright spot (i.e., array present). Of the 65.6% progenitor cells having a localized bright spot, 15.9% pass the array to only one of the daughter cells.

**Table 2:** Apparent loss rate per cell division: FU sister cells. -/- : cell had lost the array; +/- : loss at this cell division; +/+: no loss at this cell division. Loss rate is [+/-]/[(+/+) +(+/-)]. Strain PS3427 (Table 1), integrated GFP-LacI. n = 67 animals.

**GFP**				
			
**F**	**U**	**no**.	**FU %**	**Loss Rate**
+	+	37	55.2%	15.9%
-	-	23	34.3%	
+	-	7	10.4%	

### GFP-LacI • *lacO *as a single cell marker

The *ncl-1(e1865) *mutation results in enlarged nucleoli of a large number of cell types (the Ncl phenotype). Since *ncl-1 *acts in a cell autonomous manner, it is useful as a cell lineage marker [10]. The cosmid clone (C33C3) rescues the *ncl-1 *phenotype and has been used as a cell lineage marker on extrachromosomal arrays [28-30]. To test the utility of the GFP-LacI•*lacO *technique as a single cell marker, we compared it with *ncl-1*. A DNA mixture composed of the *lacO *repeat, C33C3, and *lin-15B (+) *DNA was injected into the gonads of *syIs46; ncl-1(e1865); dpy-20 (e1282); lin-15(e1763) *animals. The isolated transgenic mosaic animals were then heat shocked (See Methods). We scored the Ncl phenotype in the nucleoli of 22 different cells (m2, m3L, m3VL, m4, m3R, m3VR, m3DR, m3DL, hyp7 dorsal head, hyp7 ventral head, P/I, p3.p, P4.p, P5.p, P6, p, P7.p, P8.p, B, F, U, hyp7 ventral tail, hyp7 anus) per mosaic animal (n = 37; Table 3). The presence of the spot is unequivocal evidence that the transgene is present. The existence of Ncl cells with fluorescent spots thus clearly indicates a false negative by Ncl-1. Conversely, non-Ncl cells without a fluorescent spot suggests either the perdurance of the NCL-1 protein (i.e., false positive) or a false negative by GFP-LacI•*lacO*. Both markers agree in scoring approximately 80%, while false negatives of Ncl-1 (N, +) occur about 16% (Table 3). The occurrence of false positives of Ncl-1 (W, -) is 2%. However, neither marker is perfect. The Ncl marker is undetectable in intestinal nucleoli and endogenously large nucleoli such as some hyp cells and muscle cells. The GFP-LacI•*lacO *system has a higher apparent loss rate per cell division (15.9%; Table 3; discussed later) as well as bleaching of GFP.

**Table 3:** *ncl-1 *versus GFP-LacI•*lacO *as markers for mosaic analysis.

	**Phenotype of cells**			
	
**Cell**	**W, + or N,-**	**W, -**	**N, +**	**?**
m2	32 (87%)	0	3 (8%)	2
m3L	31 (84%)	2 (5%)	4 (11%)	
m3VL	31 (84%)	1 (3%)	5 (13%)	
m4	28 (76%)	0	9 (24%)	
m3R	31 (84%)	2 (5%)	4 (11%)	
m3VR	30 (81%)	1 (3%)	6 (16%)	
m3DR	29 (78%)	0	8 (22%)	
m3DL	30 (81%)	0	7 (19%)	
hyp7(dorsal head)	30 (81%)	1(3%)	6 (16%)	
hyp7 (ventral head)	32 (87%)	0	5 (13%)	
P/I	33 (89%)	1 (23%)	3 (8%)	
P3.p	30 (81%)	1 (3%)	3 (8%)	3
P4.p	29 (78%)	2 (5%)	4 (11%)	2
P5.p	30 (81%)	2 (5%)	4 (11%)	1
P6.p	32 (87%)	0	4 (11%)	1
P7.p	33 (89%)	0	3 (8%)	1
P8.p	26 (70%)	1 (3%)	9 (24%)	1
B	22 (59%)	1 (3%)	14 (38%)	
F	31 (84%)	1 (3%)	5 (13%)	
U	29 (78%)	2 (5%)	6 (16%)	
hyp7 (ventral tail)	29 (78%)	0	7 (19%)	1
hyp7 (anus)	21 (57%)	1 (3%)	8 (22%)	7

Mean %	80%	2%	16%	2%
Standard Dev	8%	2%	7%	4%

Mosaic animals from strain PS3047 (n = 37) were used to determine the occurrence of the Ncl phenotype and GFP-LacI•*lacO*. Scored by cell from anterior to posterior of the worm: m2, m3L, m3VL, m4, m3R, m3VR, m3DL, m3DR, hyp7(dorsal, head), hyp7(ventral head), P/I, P3.p, P4.p, P5.p, P6.p, P7.p, P8.p, B, F, U, hyp7(tail), hyp7(anus). **+**: GFP-LacI bound to the *lacO*, fluorescent spot; **-**: diffuse fluorescence, **W**: normal nucleolus (non-Ncl) size, **N**: enlarged nucleolus (Ncl) and ?: undetermined.

One explanation for the lack of intense fluorescent spots (false negatives by GFP-LacI•*lacO*) is that the expression of GFP-LacI is insufficient. We thought this possibility likely since the expression initially depended on the GFP filter combination used, and the light source (200 watt vs. 100 watt HBO burner). To increase the sensitivity, we engineered a GFP-LacI under the control of the ubiquitously expressed *dpy-30*enhancer/promoter [31]. Another possibility is that non-Ncl nuclei without spots reflect perdurance of NCL-1.

The combined use of the cell lineage marker *ncl-1 *and the GFP-LacI•*lacO *would increase the accuracy and ease of mosaic analysis. In order to use both markers, the double mutant *ncl-1; dpy-20 *was injected with the DNA mixture (pMH86 (*dpy-20(+) *+ pPD49-78::GFP-LacI + *dpy-30*::GFP-LacI). Once non-Dpy transformants were isolated, the extrachromosomal array was integrated into the genome by X-ray irradiation to yield strain PS2958 *syIs46 *[pMH86 + pPD49-78::GFP-LacI + *dpy-30::*S65T*LacI] *II; ncl-1 III; dpy-20 IV*. This strain became the basis for our genetic mosaic analysis of *lin-3*, discussed below.

The comparison between *ncl-1 *and the GFP-LacI•*lacO *as single cell markers indicate that GFP-LacI•*lacO *is comparable in its reliability to *ncl-1 *as a cell lineage marker for the presence of a transgene (Table 3). The main advantage of using the fusion protein GFP-LacI•*lacO *rather than Ncl is that scoring the mutant Ncl phenotype is typically more difficult than scoring cells with the bound GFP-LacI fusion protein. Also, GFP-LacI can be used in cells such as intestinal cells for which *ncl-1 *is not applicable. We find that the GFP-LacI method (spot or not spot) is much easier than scoring nucleolus size. Such ease of scoring may also be used for more accurate mosaic analysis. The comparison between the GFP-LacI•*lacO *and *ncl-1 *as a single cell marker as well as its loss rate per cell division (Tables 2 and 3) confirms that using both the Ncl-1 phenotype and the integrated GFP-LacI together will increase the accuracy of mosaic analysis. An alternative mosaic marker, the nuclear-localized SUR-5-GFP, demonstrates the ease and speed of scoring cells by fluorescence comparative to the *ncl-1 *marker [30]. This technique allows to rapidly screen with a dissecting microscope for rare mosaic animals, unlike the GFP-LacI•*lacO *methodology that requires a compound microscope. SUR-5-GFP is an excellent mosaic marker for determining if the gene of interest is either in the AB or P1 lineage but for finer single cell analysis, either *ncl-1 *or GFP-LacI•*lacO *is required. Another limitation of SUR-5-GFP is its limited expression pattern. GFP-LacI•*lacO *is expressed throughout the somatic cell lineage.

The high apparent loss rate of GFP-LacI•*lacO*, for example 15.9% per cell division in one experiment, could result from two general causes. First, the presence of *lacO *repeats might lead to decreased stability of the arrays. Second, the presence of the repeats might increase silencing of the transgenes in some manner [32]. We also believe that the ability to detect rapidly the presence of the transgene in individual cells affords a more accurate picture of the degree of mosaicism than gross phenotypic description.

### Polyploidy

**Figure 2:**
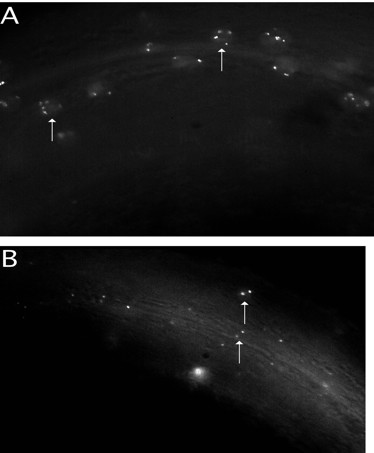
**Assay for polyploidy of nuclei**. Comparison of body muscle between *syIs44 *[*dpy-20(+)*+pPD49-78::GFPlacI+*lacO*]; *dpy-20 *and *lin-5(e1348), dpy-10(e128) II*; *syEx207 *[pRF4 *(rol-6(su1006)*) +pPD49-78GFP-LacI+*lacO*]. Animals were mounted on 5% Noble agar containing ~100 nM levamisole and examined under Nomarski microscopy and fluorescence at 100X. a) *lin-5(e1348), dpy-10(e128) II*; *syEx207 *L4 hermaphrodite. b)*syIs44*; *dpy-20 *L4 hermaphrodite. Arrows point to GFP-LacI bound to *lacO*.

Our method for ploidy detection was tested by injecting a DNA mixture consisting of a fusion protein under the control of the heat-shock promoter pPD49-78, the *lacO *repeat, plus *rol-6(su1006) *dominant into a *lin-5(e1348) *mutant, which fails to undergo mitosis [33]. The Rol segregants of strain PS2629 confirmed that GFP-LacI +*lacO *could detect polyploid cells. Multiple dots have been seen when viewing known polyploid cells (i.e., intestines and hypodermal cells; Figure 2). We conclude that the GFP-LacI•*lacO *method is useful in determining which cells are polyploid. However, the number of spots within the cell cannot unambiguously determine the extent of ploidy. In particular, diploid cells have 1–3 spots while polyploid cells have ≥ 4 spots.

The number of fluorescent spots per nucleus does not correlate precisely with the expected copy number, raising the possibility that there is some synapsis of chromosomes at some stages of the cell cycle. Nonetheless, while examination of a single cell is not sufficient, examination of several cells would be, and thus this method could be used for screens or as an assay for alterations in ploidy.

### GFP-LacI•*lacO *as a mosaic marker

As a test of this method of mosaic analysis, we chose to determine the site of action of *lin-3 *for its role in vulval induction. *lin-3 *was proposed to act in the anchor cell based upon its expression in the anchor cell, and its expression under control of heat shock enhancer/promoter could compensate for lack of an anchor cell[14,21-23]. We used a strain heterozygous for two mutant alleles of *lin-3*: *n378*, which is defective only in vulval development and *n1059 *(a genetically-defined null allele) in order to decrease *lin-3 *activity in vulval induction but still have viable animals [14,34,35]. A strain of genotype *syIs46; lin-3(n378) let-59(s49) unc-22(s7)/lin-3(n1059) unc-24(e138) *was injected with *lin-3(+) *(20 ng/μl), *lacO *(50 ng/μl), transformation marker pPD118-33 (*myo-2::GFP*) (16 ng/μl)[36], and carrier DNA BSK+II (120 ng/μl).

We examined animals with a fluorescent pharynx due to expression of MYO-2::GFP. These animals have the transgene in either the AB lineage or P1 lineage or both [36]. We picked L3–L4 animals expressing *myo-2::GFP *by viewing them under a dissecting microscope with a GFP filter. These animals were then heat-shocked for 30 minutes in a 33°C water-bath followed by a one-hour recovery period in a 20°C incubator.

**Figure 3:**
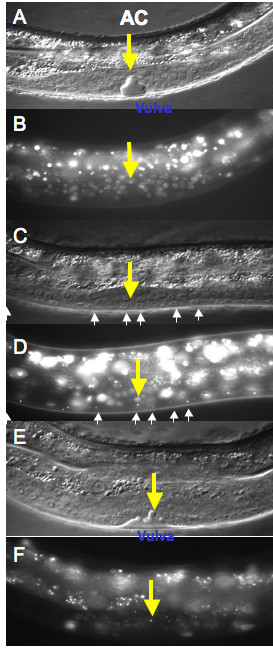
***lin-3 *mosaic analyis**. (A) *lin-3 *mosaic animal at L4 stage with normal vulval development. Anchor cell labeled with arrowhead; bar indicates vulva. (B) Same *lin-3 *mosaic animal viewed under epifluoresence. The anchor cell has an intense spot of fluorescence, and thus the transgenic array (*lin-3(+) *+ *lacO *+ *pPD118-33*). The vulval descendants do not have the intense spot of fluorescence, and thus lack the transgenic array. (C) *lin-3 *mosaic animal at L4 stage with no Pn.px cells adopting vulval fate. Anchor cell labeled with arrowhead. Pn.px cell progeny labeled with arrows. (D) Same *lin-3 *mosaic animal viewed under epifluoresence. The anchor cell does not have the intense spot of fluorescence, and thus lacks the transgenic array (*lin-3(+) *+ *lacO *+ *pPD118-33*). The Pn.px cells do have the intense spot of fluorescence, and thus have the transgenic array. (E) *lin-3 *mosaic animal at L4 stage with multivulva phenotype. Anchor cell labeled with arrowhead; bar indicates vulva. (F) Same *lin-3 *mosaic animal viewed under epifluoresence. The anchor cell has an intense spot of fluorescence, and thus the transgenic array. The vulval descendants do not have the intense spot of fluorescence, and thus lack the transgenic array.

We examined a total of 114 animals prescreened under a dissecting stereomicroscope. We considered three possibilities: *lin-3 *acts in the AC, it acts in the VPCs or both since *lacZ *and GFP reporter gene constructs indicate *lin-3 *expression in the anchor cell at the time of vulval induction [14,21-23], and that it acts in the 1° vulval lineage after vulval induction [24,25]. We scored the AC and the VPCs of wild type, Vul and Muv *lin-3 *transgenic animals. Of the 114 animals, 91 had wild-type vulva, 15 animals were vulvaless and eight animals were multivulva. Eighty-eight animals with the array present in both the AC and VPCs were wild type or Muv. All eight animals lacking the array in both the AC and VPCs were Vul, indicating that *lin-3 *is necessary either in the anchor cell, the VPCs or both. Of nine animals that had the array in the AC but not in the VPCs, eight were wild type and one was Muv (Table 4, Figure 3A–D), indicating that expression of *lin-3 *in the anchor cell is sufficient for vulval induction. All seven animals that had the array in the VPCs but not in the AC were Vul (Table 4 and Figure 3E–F), indicating that expression of *lin-3 *in the VPCs is not sufficient to induce the vulva. We conclude that *lin-3 *acts in the AC during vulval induction. Seven of sixteen animals (W.26, W.58, W.64, W.84, V.2, V.7 and V.10) had late losses during Pn.px divisions resulting in a complex mosaic pattern. We conclude that a subset of cells involved in vulva formation having the *lin-3 *gene can neither induce nor hinder vulval induction.

**Table 4:** Mosaic analysis of *lin-3 *with GFP-LacI•*lacO*

**Worm**	**P3.p**	**P4.p**	**P5.p**	**P6.p**	**P7.p**	**P8.p**	**Anchor Cell**
**W.26**	-	+	+	-	+	+	+
**W.36**	+	+	+	-	+	+	+
**W.39**	-	-	-	-	-	-	+
**W.53**	+	-	+	-	+	-	+
**W.58**	-	+	-	-	-	-	+
**W.64**	-	+	+	-	+	-	+
**W.84**	-	-	+	-	-	-	+
**W.91**	-	-	-	-	-	-	+

**V.2**	-	-	-	-	-	+	-
**V.3**	+	+	+	+	+	+	-
**V.7**	+	+	-	+	-	-	-
**V.9**	+	+	+	+	+	+	-
**V.10**	-	+	+	+	+	+	-
**V.12**	+	+	+	+	+	+	-
**V.15**	+	+	+	+	+	+	-

**M.5**	-	-	-	-	-	-	+

Mosaic analysis of *lin-3(n378)/(n1059) *animals at mid-L4 using GFP-LacI•*lacO *as the marker reveals that the wild-type *lin-3 *gene is required in the AC for normal vulval induction. **+**: GFP-LacI†*lacO*, fluorescent spot; **-**: diffuse fluorescence. Letters correspond to vulval status: **W**= normal vulva; **M**= multivulva; **V **= vulvaless. +, one of more of the indicated cell's progeny had fluorescent spots. The animal W.84 was scored at early L4.

## Conclusion

We demonstrated that GFP-LacI•*lacO *can be used to visualize transgenes in living *C. elegans*, either as extrachromosomal arrays or as arrays integrated into a chromosome. We also showed GFP-LacI•*lacO *as a useful marker for ploidy determination *in vivo*. In addition, we showed that this direct visualization using transgenes facilitate the high-resolution of mosaic analysis. The ease and accuracy of detecting mosaics using GFP-LacI•*lacO *were examined by comparing its efficiency with *ncl-1 *as a single cell marker. We demonstrated the utility of GFP-LacI•*lacO *as a mosaic marker by demonstrating that the *lin-3 *site of action for vulval induction is in the anchor cell. Overall, GFP-LacI•*lacO *is a useful tool for several aspects of *C. elegans *molecular genetics. Indeed, our system have been successfully used in several experiments by others to co-localize proteins binding DNA and for determination of ploidy [37-43].

## Methods

### Nematode methods

Growth and handling of *C. elegans *strain N2 were according to Brenner [44]. All experiments were performed at about 20°C unless otherwise stated. The genetic and cellular nomenclature of *C. elegans *was followed according to [45] and [17], respectively.

### Strains

The standard wild-type N2 strains and other mutant strains used (*ncl-1(e1865) *and *lin-5(e1348) dpy-10(e128) II*) was obtained from the *Caenorhabditis *Genetics Center (USA).

The transgene *syEx [(dpy-20(+) (20 ng/μl) + pPD49-78 GFP-LacI (100 ng/ml) + lacO (50 ng/μl)] *was integrated into a *dpy-20(e1282)IV *strain via X-ray irradiation [46]to yield PS2442 with *syIs44 *(Table 1). The transgene syEx [dpy-20(+) (20 ng/μl) +dpy-30::S65T*LacI (100 ng/μl) + pPD49-78 GFP-LacI (100 ng/μl)] was integrated into a *ncl-1; dpy-20(e1282)IV *strain via X-ray irradiation [46] to yield PS2958 with *syIs46 *(Table 1).

### GFP-LacI fusion and 256 *lacO *repeat

The GFP-LacI fusion protein and the *lacO *repeat [6-8] were graciously given to us by Andrew Belmont. We placed GFP-LacI under the transcriptional control of an *hsp16 *promoter/enhancer element pPD49-78 [47,48] The fusion protein was inserted into KpnI/SacI site of heat shock vector pPD49-78. pPD49-78 is expressed very well in the neural and hypodermal cells, as well as in the gut, muscles, and pharynx but not in the germline [47,48]. The GFP-LacI coding sequences were also placed under the transcriptional control of the *dpy-30 *promoter graciously provided by Barbara Meyer. The *dpy-30 *promoter/enhancer directs expression throughout the animal's somatic cells. [31]. The GFP was replaced with GFP(S65T) from the vector pPD93-65, which contains introns, in order to increase translation of the fusion protein [47]. The GFP (now designated S65T*) is inserted in the KpnI/EcoRI site of the *dpy-30*::GFP-LacI plasmid, now called *dpy-30*::S65T*-LacI.

### Germline-mediated transformation by microinjection

Microinjection was performed according to Mello *et al*. ([26]). Young adult hermaphrodites were placed live on pads of 2% agarose under an inverted differential contrast-interference (Nomarski) microscope (Carl Zeiss, Oberkochen, West Germany) and the DNA was injected into the gonad using an Eppendorf micro injector 5242 (Eppendorf Gertebau Netheler, Hamburg, West Germany). The Ncl vs. GFP-LacI experiments was done in a *syIs46; lin-15(e1763) *background. The injection mixture for the Ncl vs. GFP-LacI experiment contained the 256 repeat *lacO *array (50 ng/μl), cosmid C33C3 (rescues the Ncl-1 mutant phenotype [29,49] (50 ng/μl), *lin-15B(+) *genomic DNA (50 ng/μl) and pBluescript II SK+ (Stratagene) as carrier DNA (5 ng/μl). For the polyploidy experiments, the plasmid pRF4, containing the *rol-6(su1006) *mutant gene [26], was used as a dominant transformation marker at a concentration of 40 ng/μl. The injection mixture for the polyploidy experiments contained pPD49-78::GFP-LacI (100 ng/μl), 256 repeat *lacO *array (50 ng/μl), and pBluescript II SK+ (Stratagene) as carrier DNA (5 ng/μl). This injection mixture was injected into the gonads of *lin-5(e1348) dpy-10(e128) II *to yield *syEx207 *in the strain PS2629.

The transgenic lines obtained from each experiment were heat-shocked for 30 minutes at 33°C to elicit GFP-LacI expression. Expression of the GFP-LacI can be seen as early as 30 minutes after heat-shock, and as late as 24 hours.

### Mosaic analysis of *lin-3 *gene function in vulval induction

Mosaic animals were obtained from a somatic loss of the extrachromosomal array *syEx345 [lin-3(+), lacO, myo-2::GFP(pPD118-33)] *from the vulvaless strain PS3427 *syIs46; lin-3(n378) let-59(s49) unc22(s7)/lin-3(n1059)unc-24(e138) *(Table 1). The point of loss was determined by the absence of fluorescent spots in cells of interest. For mosaic analysis, we used L3–L4 wild-type, multivulva, and vulvaless animals with their pharynges fluorescing due to expression of MYO-2::GFP [36] to ensure the array was present in the zygote. The nuclei observed to identify mosaic animals were: AC, P3.p, P4.p, P5.p, P6.p, P7.p and P8.p, or the descendants of these latter six cells.

### Microscopy and photography

Animals were anesthetized with 2 mM levamisole on 5% Noble agar pads. Photographs were taken on Kodak Ektachrome, ASA 160 or Fuji Provia, ASA 400 on a Zeiss Axioplan with Chroma High Q GFP LP filter set (absorption band 450 nm and 505 nm emission) at 100 X optics or by confocal photomicrography for strain PS2442.

## Abbreviations

Is, Insertion Site (integrated transgene)

GFP, green fluorescent protein

## Authors' contributions

AG-S designed and executed all the experiments. AG-S and PWS analyzed the data and wrote the paper.

## References

[B1] Chalfie M, Tu Y, Euskirchen G, Ward WW, Prasher DC (1994). Green fluorescent protein as a marker for gene expression. Science.

[B2] Cubitt AB, Heim R, Adams SR, Boyd AE, Gross LA, Tsien RY (1995). Understanding, improving and using green fluorescent proteins. Trends Biochem Sci.

[B3] Heim R, Prasher DC, Tsien RY (1994). Wavelength mutations and posttranslational autoxidation of green fluorescent protein. Proc Natl Acad Sci U S A.

[B4] Heim R, Tsien RY (1996). Engineering green fluorescent protein for improved brightness, longer wavelengths and fluorescence resonance energy transfer. Curr Biol.

[B5] Belmont AS, Straight AF (1998). In vivo visualization of chromosomes using lac operator-repressor binding. Trends Cell Biol.

[B6] Robinett CC, Straight A, Li G, Willhelm C, Sudlow G, Murray A, Belmont AS (1996). In vivo localization of DNA sequences and visualization of large-scale chromatin organization using lac operator/repressor recognition. J Cell Biol.

[B7] Straight AF, Belmont AS, Robinett CC, Murray AW (1996). GFP tagging of budding yeast chromosomes reveals that protein-protein interactions can mediate sister chromatid cohesion. Curr Biol.

[B8] Webb CD, Decatur A, Teleman A, Losick R (1995). Use of green fluorescent protein for visualization of cell-specific gene expression and subcellular protein localization during sporulation in Bacillus subtilis. J Bacteriol.

[B9] Yochem J, Herman RK, Community TCR (2005). Genetic Mosaics. WormBook.

[B10] Hedgecock EM, Herman RK (1995). The ncl-1 gene and genetic mosaics of Caenorhabditis elegans. Genetics.

[B11] Herman RK (1984). Analysis of genetic mosaics of the nematode Caneorhabditis elegans. Genetics.

[B12] Herman RK, Hedgecock EM (1990). Limitation of the size of the vulval primordium of Caenorhabditis elegans by lin-15 expression in surrounding hypodermis. Nature.

[B13] Miller LM, Gallegos ME, Morisseau BA, Kim SK (1993). lin-31, a Caenorhabditis elegans HNF-3/fork head transcription factor homolog, specifies three alternative cell fates in vulval development. Genes Dev.

[B14] Hill RJ, Sternberg PW (1992). The gene lin-3 encodes an inductive signal for vulval development in C. elegans. Nature.

[B15] Sternberg PW, Community TCR (2005). Vulval development. WormBook,.

[B16] Sternberg PW, Horvitz HR (1986). Pattern formation during vulval development in C. elegans. Cell.

[B17] Sulston JE, Horvitz HR (1977). Post-embryonic cell lineages of the nematode, Caenorhabditis elegans. Dev Biol.

[B18] Katz WS, Hill RJ, Clandinin TR, Sternberg PW (1995). Different levels of the C. elegans growth factor LIN-3 promote distinct vulval precursor fates. Cell.

[B19] Simske JS, Kim SK (1995). Sequential signalling during Caenorhabditis elegans vulval induction. Nature.

[B20] Sundaram MV (2005). RTK/Ras/MAPK signaling. WormBook.

[B21] Kimble J (1981). Alterations in cell lineage following laser ablation of cells in the somatic gonad of Caenorhabditis elegans. Dev Biol.

[B22] Wang M, Sternberg PW (1999). Competence and commitment of Caenorhabditis elegans vulval precursor cells. Dev Biol.

[B23] Hwang BJ, Sternberg PW (2004). A cell-specific enhancer that specifies lin-3 expression in the C. elegans anchor cell for vulval development. Development.

[B24] Chang C, Newman AP, Sternberg PW (1999). Reciprocal EGF signaling back to the uterus from the induced C. elegans vulva coordinates morphogenesis of epithelia. Curr Biol.

[B25] Dutt A, Canevascini S, Froehli-Hoier E, Hajnal A (2004). EGF signal propagation during C. elegans vulval development mediated by ROM-1 rhomboid. PLoS Biol.

[B26] Mello CC, Kramer JM, Stinchcomb D, Ambros V (1991). Efficient gene transfer in C.elegans: extrachromosomal maintenance and integration of transforming sequences. Embo J.

[B27] Yochem J, Hall DH, Bell LR, Hedgecock EM, Herman RK (2005). Isopentenyl-diphosphate isomerase is essential for viability of Caenorhabditis elegans. Mol Genet Genomics.

[B28] Koga M, Ohshima Y (1995). Mosaic analysis of the let-23 gene function in vulval induction of Caenorhabditis elegans. Development.

[B29] Miller LM, Waring DA, Kim SK (1996). Mosaic analysis using a ncl-1 (+) extrachromosomal array reveals that lin-31 acts in the Pn.p cells during Caenorhabditis elegans vulval development. Genetics.

[B30] Yochem J, Gu T, Han M (1998). A new marker for mosaic analysis in Caenorhabditis elegans indicates a fusion between hyp6 and hyp7, two major components of the hypodermis. Genetics.

[B31] Hsu DR, Chuang PT, Meyer BJ (1995). DPY-30, a nuclear protein essential early in embryogenesis for Caenorhabditis elegans dosage compensation. Development.

[B32] Kelly WG, Xu S, Montgomery MK, Fire A (1997). Distinct requirements for somatic and germline expression of a generally expressed Caernorhabditis elegans gene. Genetics.

[B33] Albertson DG, Sulston JE, White JG (1978). Cell cycling and DNA replication in a mutant blocked in cell division in the nematode Caenorhabditis elegans. Dev Biol.

[B34] Ferguson EL, Horvitz HR (1985). Identification and characterization of 22 genes that affect the vulval cell lineages of the nematode Caenorhabditis elegans. Genetics.

[B35] Liu J, Tzou P, Hill RJ, Sternberg PW (1999). Structural requirements for the tissue-specific and tissue-general functions of the Caenorhabditis elegans epidermal growth factor LIN-3. Genetics.

[B36] Okkema PG, Harrison SW, Plunger V, Aryana A, Fire A (1993). Sequence requirements for myosin gene expression and regulation in Caenorhabditis elegans. Genetics.

[B37] Carmi I, Kopczynski JB, Meyer BJ (1998). The nuclear hormone receptor SEX-1 is an X-chromosome signal that determines nematode sex. Nature.

[B38] Csankovszki G, McDonel P, Meyer BJ (2004). Recruitment and spreading of the C. elegans dosage compensation complex along X chromosomes. Science.

[B39] Dawes HE, Berlin DS, Lapidus DM, Nusbaum C, Davis TL, Meyer BJ (1999). Dosage compensation proteins targeted to X chromosomes by a determinant of hermaphrodite fate. Science.

[B40] Lieb JD, de Solorzano CO, Rodriguez EG, Jones A, Angelo M, Lockett S, Meyer BJ (2000). The Caenorhabditis elegans dosage compensation machinery is recruited to X chromosome DNA attached to an autosome. Genetics.

[B41] Chu DS, Dawes HE, Lieb JD, Chan RC, Kuo AF, Meyer BJ (2002). A molecular link between gene-specific and chromosome-wide transcriptional repression. Genes Dev.

[B42] Yonker SA, Meyer BJ (2003). Recruitment of C. elegans dosage compensation proteins for gene-specific versus chromosome-wide repression. Development.

[B43] Kaltenbach L, Horner MA, Rothman JH, Mango SE (2000). The TBP-like factor CeTLF is required to activate RNA polymerase II transcription during C. elegans embryogenesis. Mol Cell.

[B44] Brenner S (1974). The genetics of Caenorhabditis elegans. Genetics.

[B45] Horvitz HR, Brenner S, Hodgkin J, Herman RK (1979). A uniform genetic nomenclature for the nematode Caenorhabditis elegans. Mol Gen Genet.

[B46] Fire A (1986). Integrative transformation of Caenorhabditis elegans. Embo J.

[B47] Mello C, Fire A (1995). DNA transformation. Methods Cell Biol.

[B48] Perry MD, Li W, Trent C, Robertson B, Fire A, Hageman JM, Wood WB (1993). Molecular characterization of the her-1 gene suggests a direct role in cell signaling during Caenorhabditis elegans sex determination. Genes Dev.

[B49] Frank DJ, Roth MB (1998). ncl-1 is required for the regulation of cell size and ribosomal RNA synthesis in Caenorhabditis elegans. J Cell Biol.

